# Development of PCA-MLP Model Based on Visible and Shortwave Near Infrared Spectroscopy for Authenticating Arabica Coffee Origins

**DOI:** 10.3390/foods12112112

**Published:** 2023-05-24

**Authors:** Agus Dharmawan, Rudiati Evi Masithoh, Hanim Zuhrotul Amanah

**Affiliations:** Department of Agricultural and Biosystems Engineering, Faculty of Agricultural Technology, Universitas Gadjah Mada, Bulaksumur, Yogyakarta 55281, Indonesia; agusdharmawan@mail.ugm.ac.id (A.D.); hanim_za@ugm.ac.id (H.Z.A.)

**Keywords:** Arabica coffee, authentication, spectroscopy, principal component analysis, multilayer perceptron

## Abstract

Arabica coffee, one of Indonesia’s economically important coffee commodities, is commonly subject to fraud due to mislabeling and adulteration. In many studies, spectroscopic techniques combined with chemometric methods have been massively employed in classification issues, such as principal component analysis (PCA) and discriminant analyses, compared to machine learning models. In this study, spectroscopy combined with PCA and a machine learning algorithm (artificial neural network, ANN) were developed to verify the authenticity of Arabica coffee collected from four geographical origins in Indonesia, including Temanggung, Toraja, Gayo, and Kintamani. Spectra from pure green coffee were collected from Vis–NIR and SWNIR spectrometers. Several preprocessing techniques were also applied to attain precise information from spectroscopic data. First, PCA compressed spectroscopic information and generated new variables called PCs scores, which would become inputs for the ANN model. The discrimination of Arabica coffee from different origins was conducted with a multilayer perceptron (MLP)-based ANN model. The accuracy attained ranged from 90% to 100% in the internal cross-validation, training, and testing sets. The error in the classification process did not exceed 10%. The generalization ability of the MLP combined with PCA was superior, suitable, and successful for verifying the origin of Arabica coffee.

## 1. Introduction

Coffee (*Coffea* sp.), one of the most important national plantation commodities, is critical to Indonesia because it boosts foreign exchange and social welfare. In 2019, coffee was grown on state plantations (14.5 thousand ha), private plantations (9.71 thousand ha), and smallholder plantations (1.215 million ha), with a total production of 741,657 tons. A total of 359,052 tons of coffee were exported abroad with foreign exchange earnings of USD 883 million [1]. The five provinces of Sumatra Island, namely South Sumatra, Lampung, Aceh, North Sumatra, and Bengkulu, were the top national coffee producers, followed by East Java on Java Island, and South Sulawesi on Sulawesi Island. Two species of coffee are widely cultivated due to being geographically and climatologically well-suited for growing in Indonesia: Robusta (*Coffea canephora*) and Arabica (*Coffea arabica*).

Arabica coffee is the most cultivated coffee species, accounting for roughly 70% of the global coffee market’s availability. Arabica coffee is also one of the most popular coffee beverages. It has a rich flavor, is less bitter, and contains low caffeine. Arabica coffee trees grow well at an altitude of 1000–2100 m above sea level with an air temperature of 18–22 °C and an annual rainfall of at least 1500 mm [2,3]. Several locations became major Arabica coffee production zones in Indonesia, including Aceh, North Sumatra, Sulawesi, Flores, Bali, and East Java [4]. The quality of Arabica coffee is affected by a variety of factors, including cultivar genetics, agro-climatic conditions, agricultural practice management, and postharvest processing. The growing locations of Arabica coffee determine its quality regarding physical aspects and chemical composition [5]. Coffee quality is evaluated based on chemical, organoleptic, and physical attributes. Chemical assessments of coffee beans are complex, owing to the wide range of chemical compounds (nonvolatile and volatile) formed and contained [6]. Organoleptic properties are related to the aroma, flavor, sweetness, acidity, or overall taste of coffee. Shape, thickness, weight, and color are examples of physical characteristics [7].

Variability in coffee quality, taste, and body can be caused by the region where the coffee plants are grown. This variability aspect affects the commercial value of the product and has led to fraud such as mislabeling and adulteration. Mislabeling coffee means disguising the right geographical origin of coffee beans, while adulteration mixes and sells less-qualified coffee as pure-graded-expensive coffee [8]. As a result, coffee producers and industries are concerned about preserving their market reputation to overcome these issues. The examination of coffee beans becomes important to affirm the authenticity of coffee and to declare whether the coffee is, in fact, what it is declared to be or belongs to the defined geographical origin. This outcome will also mean that the coffee quality meets technical/regularity documentation [9]. In addition to the purposes of trading and purchasing, producers and industries need information that correlates to coffee quality from bean to cup (beverages) [10].

Several analytical techniques have been used to examine the authenticity of coffee dependent on its chemical composition, such as gas and liquid chromatography, mass spectrometry, and nuclear magnetic resonance spectrometry [8]. In recent studies, spectroscopic techniques have also been used to ensure accurate outcomes in evaluating the chemical composition and discrimination of agricultural products, including coffee. They are green, simple, rapid, robust, inexpensive, and nondestructive (do not need sample pretreatment) in the evaluation [8,11]. Regardless of these methods, several chemometric tools are usually needed to improve a series of classification models, such as principal component analysis (PCA) [12], hierarchical cluster analysis (HCA) [13], soft independent modeling by class analogy (SIMCA) [14], linear discriminant analysis (LDA) [15], and partial least square discriminant analysis (PLS-DA) [16]. The effective models can generate classification accuracy close to 100% [17]. The use of these methods for coffee authenticity in recent studies has gained the best accomplishment accuracy according to the geographical origins of Indonesian coffee in the form of green beans [8,18,19], roasted beans [20,21], and powder [22].

Artificial neural networks (ANNs) are classified as supervised learning in which a certain number of groups are determined based on feature data and labeled datasets trained to produce correct results [23,24]. Inspired by the functional characteristics of human brains, ANNs are able to work such complex functions, including learning, recognition, classification, and decision-making [25,26]. ANNs are a simplified model of biological neural networks, where billions of neurons are interconnected, organized, and processed any information provided. The neurons in ANNs are organized in layers, namely (1) an input layer, where the data are fed, (2) one or more hidden layers, where the learning process takes place, and (3) an output layer, where the decision is generated [25,27]. The structure of this network is a variant of the original perceptron model proposed by Rosenblat in 1950 and is mentioned as multilayer perceptron [28]. Multilayer perceptron (MLP) is a feed-forward neural network where the information is propagated through the network feed-forwardly from the input layer to the output layer [27,29]. Every neuron in a layer is connected to all neurons in the next layer (not inter-connected in the same layer). MLP also often uses a back-propagation algorithm to handle errors generated during the forward pass. The algorithm feeds the losses backwardly through the network by improving the weights and bias.

This study aimed to develop a classifier for authenticating coffee beans using a combination of a dimensional reduction technique, principal component analysis (PCA), and a nonlinear model, artificial neural network (ANN). The green coffee samples were from the same species, Arabica, and were grown in different regions of Indonesia, including Temanggung (East Java), Toraja (South Sulawesi), Gayo (Aceh), and Kintamani (Bali). Coffees from these origins were regarded as extensively cultivated in coffee plantations, top-graded, and widely exported abroad. PCA was employed to obtain the most important information, decrease the dimensionality of the spectroscopic data, and express that information as a dataset called principal components (PCs) [30]. The PCs scores were used as input for the MLP model. An ANN based on multilayer perceptron (MLP) was used in this study, which was a powerful learning system with superior pattern recognition ability [24,25]. The use of an MLP model to discriminate food and agricultural products dependent on spectroscopic techniques has been reported in several studies [25,26]. Two spectrometers will be used in this work, including a visible to near-infrared (Vis–NIR) spectrometer (400–1000 nm) and a shortwave near-infrared (SWNIR) spectrum (970–1630 nm). Several spectral pretreatment methods were employed to lessen noise and remove the light scattering effect in raw spectra [31]. 

## 2. Materials and Methods

### 2.1. Material Preparation

Arabica green beans were purchased from trusted local markets in Indonesia and harvested in 2022. The samples were collected from various locations, including Temanggung (Middle Java), Toraja (South Sulawesi), Gayo (Aceh), and Kintamani (Bali). All beans were from full-washed coffee processing. The beans (100 g each) were cleaned manually to remove endocarp/parchment and dirt and separate them from uniform and damaged beans. Before the spectral acquisition, the samples were placed in plastic boxes at controlled temperature of 25–28 °C to maintain the coffee quality.

### 2.2. Spectral Acquisition and Pre-Processing

The reflectance spectra were obtained using a Vis–NIR spectrometer (Flame-T-VIS–NIR Ocean optics, Orlando, FL, USA, 400–1000 nm) and an NIR spectrometer (Flame-NIR Ocean optics, Orlando, FL, USA, 970–1630 nm). A tungsten halogen light (360–2400 nm, HL-2000-HP-FHSA Ocean Optics, Orlando, FL, USA, nominal bulb power 20 W, typical output power 8.4 mW) and a reflectance fiberoptic probe (QR400-7 VIS–NIR Ocean Optics, Orlando, FL, USA) were used in both spectrometers. A black box was used during spectral measurements to eliminate light interference from external sources. The distance between the samples and the sensor probe was 5 mm. Prior to spectral acquisition of each sample, white-dark reference spectra were measured, one from a white-background ceramic (WS-1, Ocean Optics, Orlando, FL, USA) and the dark reference came from the off-light source of the instrument system. Coffee spectra were collected using OceanView 1.6.7 software (Ocean Insight, Orlando, FL, USA) with an integration time of 1600 ms and a boxcar width of 1. In order to ensure the accuracy of spectral data acquisition, both spectrometers were preheated for 15 min before testing to maintain the instrument’s internal system stability, and a self-check was carried out to see if the instrument worked normally. A total of 2400 spectral data points of green coffee beans were collected from 4 origins × 600 beans. The raw spectral data were stored in CSV format.

This study used raw and preprocessed spectra for the classification model. Several techniques were carried out to attain precise information from spectroscopic measurements [32]. The simple moving average (SMA) and Savitzky–Golay (SG) filters were employed to denoise and smooth the spectral information. The number of points to be averaged in the spectrum at the SMA filter was 50 for Vis–NIR and 5 for SWNIR. The SG smoothing (SGS) and first derivatives (SG-1D), polyorder = 2, with a window size of 50 (Vis–NIR) and 5 (SWNIR), were also used. The multiplicative scatter correction (MSC) and standard normal variate (SNV) were used to deal with scattering disturbance by eliminating baseline effects caused by translation and offset in the spectrum.

### 2.3. Data Dimensional Reduction

PCA was used to extract important information from spectroscopy data and express it as a set of new orthogonal variables known as principal components (PCs) [30]. By plotting PCs based on the characteristic wavelengths from the original and preprocessed spectra, the clustering between the different groups of samples was evaluated [33]. The evaluation of PCA was discovered through the interpretation of scree plots, scores plots, and loading plots. The scree plot interprets the variance values of individual PCs versus the PC number. In this study, it was performed on the explained variance ratio of PCs. The score plot interprets the sample coordinates projected onto the new successive axes (PCs). The PC scores with the explained variance ratio >0.5% will be used as input data for the classification model. The loading plot equates the contribution of variables in these same spaces [34].

### 2.4. Structure of Classification Model

**Figure 1:**
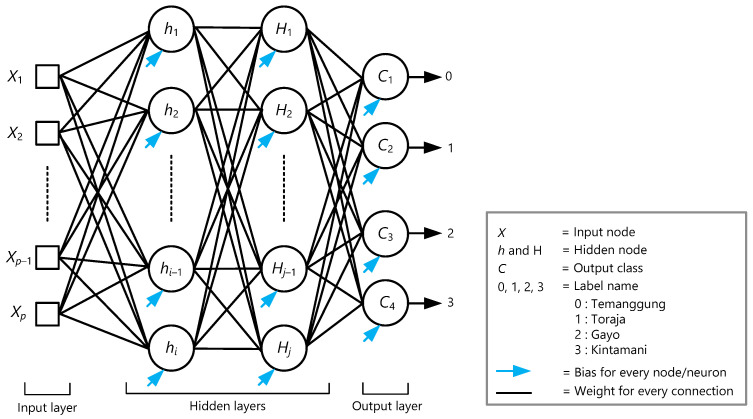
MLP architecture for multiple classifications of coffee origins.

This study developed an artificial neural network (ANN) model based on a multilayer perceptron (MLP). This model uses a sequential model to arrange all layers in sequence, it specifies a neural network, to be precise, sequential: from input to output, passing through a series of hidden layers, one after the other. The MLP in this study consisted of an input layer, two hidden layers, and an output layer. The MLP architecture is shown in Figure 1. 

This study used *p* features in the input layer (*X*); the number of *p* was obtained from PCs scores generated. In order to achieve the best classification results, the structure of the hidden layers in the MLP model was determined as *i* neurons in hidden layer 1 (*h*) and *j* neurons in hidden layer 2 (*H*). The number of *i* and *j* was determined as equal. The output layer had multiple nodes as it would classify four origins of coffee beans. With the one-hot encoding technique, each categorical value in the output layer was converted into a new categorical column and assigned a binary value of 1 or 0 to the column, 4-class classification problem: class 0 (temanggung) → [1, 0, 0, 0], class 1 (toraja) → [0, 1, 0, 0], class 2 (gayo) → [0, 0, 1, 0], class 3 (kintamani) → [0, 0, 0, 1]. 

Several parameters must also be considered during building this model, including activation function, method of weight initialization, loss function, validation method, batch size, the number of epochs, etc. All these values and parameters were defined experimentally to generate the best outcomes for the model which will also be elaborated on in the experimental results and discussion sessions.

### 2.5. K-Fold Cross-Validation

**Figure 2:**
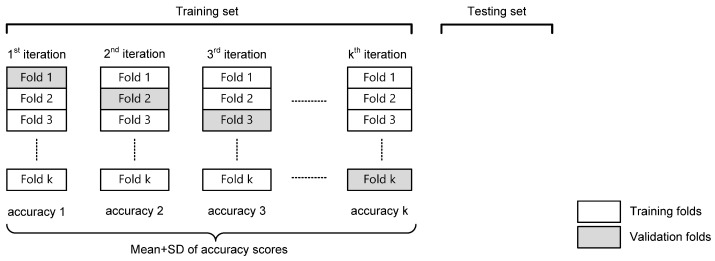
Scheme of k-fold validation process.

In this study, the dataset was split into two parts—a training set and a testing set. About 2/3 of random samples were put in the training set, and the remaining 1/3 was assigned to the testing set. K-fold cross-validation evaluates the model’s ability in certain data to classify new data and flag problems such as overfitting [35,36]. The training data were divided into *k* subsets (folds). *k* refers to the number of folds that a given dataset that will split into. This study determined *k* = 10. Since we had about 1600 training data and *k* = 10, each fold contains around 160 data. In these partitioned folds, training and testing subsets were performed in *k* iterations such that in each iteration, we put one fold for validation and left the remaining *k* − 1 folds to train the model [37,38]. The total effectiveness of the model was ascertained by calculating the average of each iteration and the estimation error generated. An illustration of this validation method is given in Figure 2.

### 2.6. Model Evaluation

The confusion matrix was used for model performance evaluation [39]. First, we must compute a set of predicted targets and compare them with actual targets [29]. The predicted targets represent the values of the class as a result of the model, while the actual class represents the original values of the initial class [40]. The general idea is to count the correct/incorrect classifications of positive samples and the correct/incorrect classification of negative samples [41]. A schematic representation of multi-class confusion matrix of coffee origin is shown in Table 1. The determination of *TP*, *TN*, *FP*, and *FN* are calculated using formulas given in Table 2. The *TP* (True Positive) values represent the number of correctly classified positive examples; the *TN* (True Negative) values estimate the number of correctly classified negative examples; the *FP* (False Positive) values represent the number of correctly classified negative examples; and the *FN* (False Negative) is the number of actual positive examples grouped as negative [35]. 

The performance of the MLP model was determined by calculating performance metrics. The most commonly employed indicators are accuracy, specificity, precision, recall or sensitivity, and F-score; the formulas are given in Table 3. Accuracy (*AC*) estimates the proportion of correctly classified samples, whereas Misclassification error (*E*) estimates the proportion of incorrectly classified samples. Specificity (*SP*) counts the ratio of incorrectly classified samples to all negative samples. Recall (R) or sensitivity calculates the ratio of correctly classified samples to all positive samples. Precision (P) measures the ratio of correctly classified samples as positive to all the positively classified samples. F-score (FS) combines the precision and recall scores of a model. While ‘accuracy’ remains valid for balanced data, F-score works well on imbalanced data. The *AC* evaluates the correct classified samples, and the values shall be close to 100%. The *SP*, *R*, *P*, and *FS* are declared as ‘good’ when they are close to 1. The *E* evaluates wrongly classified samples, which shall be as low as possible, ideally close to 0% [36].

**Figure 3:**
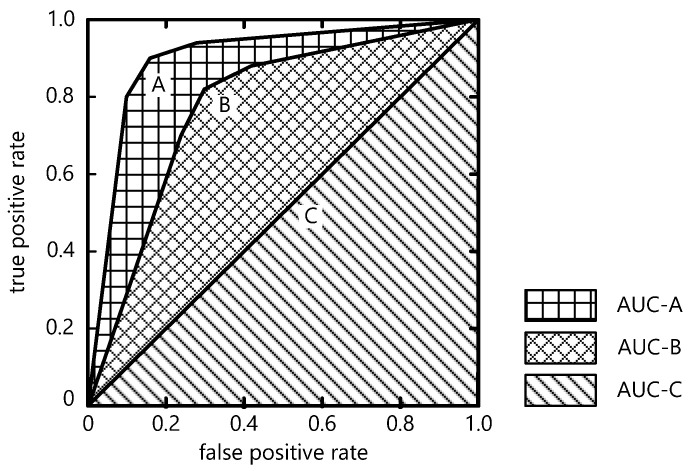
Sample of an ROC graph with three ROC curves.

The area under the curve (AUC) and receiver operating characteristic (ROC) curve were also determined to check or visualize the performance of this classification model. The ROC curve for the multi-class problems contains a graph that represents TPR (true positive rate) on the *y*-axis against FPR (false positive rate) on the *x*-axis [42,43]. The proportion of true positive comes from the value of sensitivity, while the proportion of false negative comes from 1-specificity [44]. A ROC curve starts at point (0,0) and ends at point (1,1). The point at coordinate (0,0) (TPR = 0, FPR = 0) represents that the classifier never predicts a positive class. Point (1,1) (TPR = 1, FPR = 1) represents the opposite situation, the classifier classifies all samples as positive and produces a possibly high number of false positives. The perfect coordinate is at (0,1) while TPR = 1 and FPR = 0. Figure 3 shows an example of an ROC graph with three ROC curves. Classifier A performs far better than the other two (B and C). Classifier C is useless as its performance is no better than chance [37]. 

The area under the curve (AUC) summarizes each ROC curve in the form of numerical information. The AUC is calculated by summing the area under the ROC curve; the larger the area, the more accurate the model is [44]. The AUC value lies on the interval 0 to 1. The greater the AUC value, the better the classification model. Since a better classification model should lie above the ascending diagonal of an ROC graph (curve C in Figure 3), the AUC must exceed the value of 0.5 [37].

All data analyses in this work—from spectral preprocessing to PCA analysis to the development and evaluation of the MLP model—were conducted by PyCharm 2022.3 (Professional Edition for educational use) as an IDE platform for Python code (v3.10). In addition, a number of libraries were used, such as Pandas (v2.0.0), NumPy (v1.24.2), Matplotlib (v3.7.1), SciPy (v1.10.1), Scikit-Learn (v1.2.2), and Keras (v2.11.0).

## 3. Results

### 3.1. Spectral Profiles of Arabica Coffee

**Figure 4:**
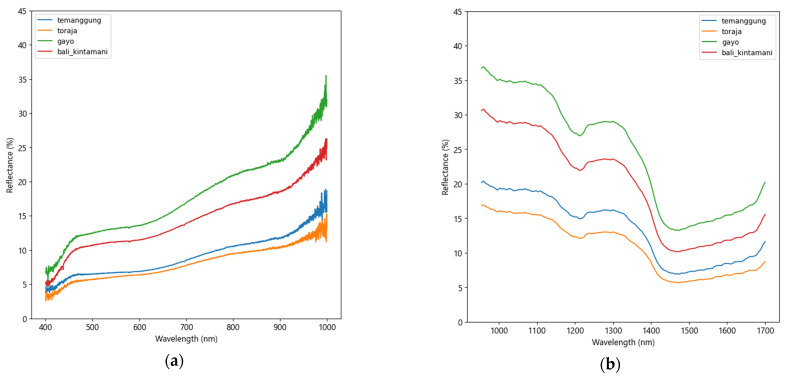
Original reflectance spectra of four Arabica coffees: (**a**) Vis–NIR and (**b**) SWNIR.

The averaged raw spectra of four Arabica beans are shown in Figure 4. Visible near-infrared (Vis–NIR) spectra ranging from 400 to 1000 nm contain 3182 variables, whereas shortwave near-infrared (SWNIR) spectra ranging from 954 to 1700 nm contain 128 variables. The spectral profiles of the four Arabica coffee origins are similar and characterized by differences in curve trends. These spectral properties are determined by how samples interact with light radiation. The light can be transmitted, absorbed, or reflected when it strikes coffee samples [45]. The amount of radiation interacting with the samples is determined by their chemical and physical properties [46].

Using original Vis–NIR spectra (Figure 4a), all coffee gives similar trends, with differences in the reflectance (or absorbance) intensities. Toraja coffee has the highest absorbance (lowest reflectance) followed by Temanggung, Kintamani, and Gayo coffee. Reflectance peaks and valleys can be noticed at 460 nm of lignin [47], 670 nm of chlorophylls [48], which are present in coffee [48]. Other low absorbance peaks appear at 750–850 nm of O-H of water [43] and 900–1000 nm of C-H groups of coffee [49]. 

Based on SWNIR spectra, all coffees show a similar pattern (Figure 4b). The highest absorbance is displayed by Toraja coffee, followed by Temanggung, Bali Kintamani, and Gayo coffee. More distinct absorption peaks are observed than Vis–NIR profiles. The fundamental absorptions at particular bands occur, including the 3rd overtone at the 900–1000 nm region, the 2nd overtone at 1100–1200 nm, and the 1st overtone at 1400–1500 nm [11]. These absorption bands have been linked to the main compounds found in coffee, including carbohydrates, lipids, proteins, caffeine, chlorogenic acid, and water [50]. According to Figure 4a,b, it is challenging to draw a conclusion and associate the spectral properties of four coffee origins because they generate similar curve trends. Therefore, further chemometric methods and machine learning models are required for the classification purpose.

### 3.2. Principal Component Analysis

Teye et al. [51] define principal component analysis (PCA) as an unsupervised pattern recognition method for visualizing data trends in dimensional space. PCA is employed in this study to analyze spectroscopic data from coffee samples in which observations are described by several dependent variables, which are generally intercorrelated. PCA will extract the important information from the data, express it as a set of new orthogonal variables known as PCs, and display a pattern of similarity between the observations and variables as points on maps [30]. In the spectroscopic analysis, new variables (called PCs) are a linear combination of the original wavelength variables and show the maximum variation contained within them [52,53]. The PC1 is a set of variables that explain the largest variance, and PC2 is independent of PC1 and defines the second-largest variance. Other PCs can be specified as well [52]. A score plot was obtained by plotting the PCs to visualize the data trends, and it explained the maximum variances or weights. These PCs weights versus PC number then explain more than 0.5% variance given in Table 4.

**Figure 5:**
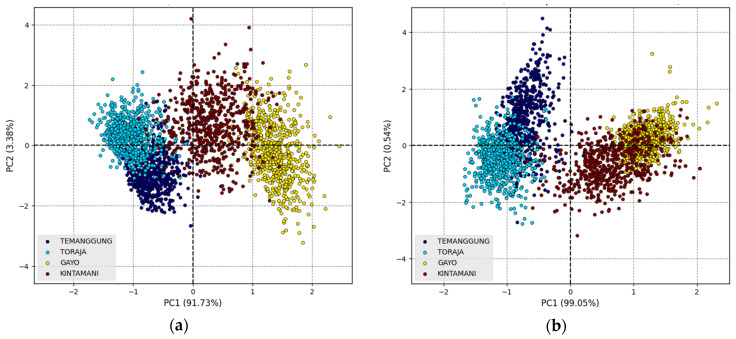
PCA score plots for original spectra: (**a**) Vis–NIR and (**b**) SWNIR.

Figure 5 shows the results of the PCs score plot from the original spectroscopic data. The sum of PC1 and PC2 can explain more than 95% of the variances for Vis–NIR and SWNIR spectra. It reveals that there are separations in the samples. The chemical properties of coffee beans differ significantly depending on their geographical origins. According to Figure 5, the first two PCs (PC1 and PC2) already accounted for >90% of the total contribution. The smoothed spectra from the moving average filter and SG smoothing produced the best outcome in explaining the maximum variance since it aimed at advancing signal quality [31]. In contrast, the maximum explained variance did not occur in the first two PCs for preprocessed spectra from SNV, MSC, and SG-1D in VisNIR spectra. The MSC and SNV try separating multiplicative interferences in the spectra, such as light scattering effects. In contrast, the SG-1D is commonly used to eliminate baseline offset variations within a set of spectra [31,54].

**Figure 6:**
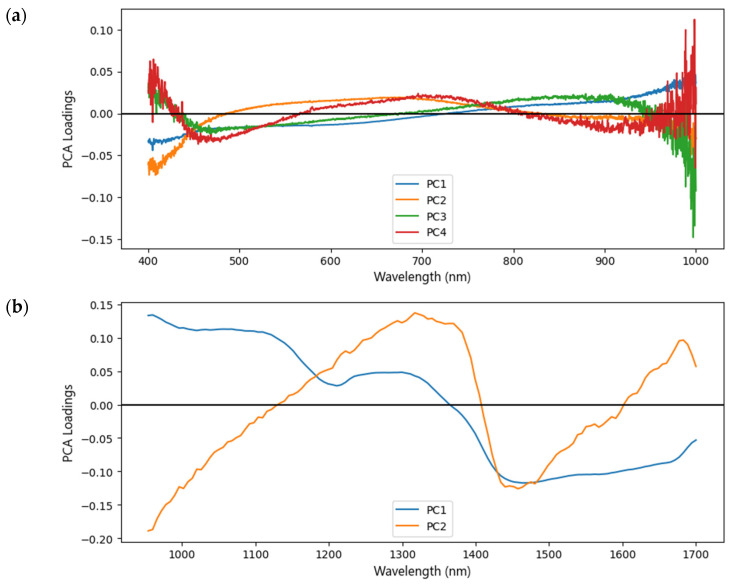
PCA loading plots from original spectra (**a**) VisNIR, (**b**) SWNIR.

The loading plots were also obtained to evaluate the areas of spectra that contribute to the variance of samples. The loading matrix includes the original variables’ contributions in the same space [34]. This contribution affects the clustering between samples [55], indicating that the chemical compositions between groups of Arabica coffee were considerably different. The loadings plot for the first PCs from the original spectroscopic data is shown in Figure 6. The second overtone of the O–H and N–H stretches was found in the small region 850–1050 (PC1,3,4) of Vis–NIR spectra [56]. At 940 nm, the spectral variable corresponds to the third overtone absorbance band of the –CH_3_– group [31]. Major valley (PC1) is found in the second overtone of C–H stretching for caffeine and carbohydrates in pure coffee at a wavelength of 1210 nm. The C–H stretching and deformation vibration for caffeine is also found at ~1300 nm. At 1450 nm, water, or the first overtone of the O–H stretch, can be found. The amino acids and chlorogenic acid are related to the first O–H, and N-overtone at ~1570 nm [57].

### 3.3. Multilayer Perceptron Model

In this study, developing a classifier was crucial to obtain the best accuracy outcome in the classification process. The MLP model was organized into layers consisting of an input, hidden, and output layers, Figure 1. The model was trained with a large number of data samples (containing inputs and outputs) based on a supervised learning technique to identify the geographical origins of Arabica coffee. Input data were obtained from the PCs score which had the explained variance ratio >0.5%. The desired outputs were considered as four coffee origins in the form of classes: class 0 for Temanggung, class 1 for Toraja, class 2 for Gayo, and class 3 for Kintamani. 

The problems regarding the hidden layer must also be considered carefully, especially with regard to how many hidden layers to put in a neural network and how many neurons will be in each of these layers. There is no theoretical reason to use one or more hidden layers [58]. However, in practice, we can start with one hidden layer to get reasonable results and gradually continue with two or more hidden layers until the model performs better [29]. For more complex ones, we can gradually ramp up the number of hidden layers until the model starts to overfit. However, it is recommended to train such networks from scratch. It is much more common to reuse parts of a pre-trained state-of-the-art network that performs a similar task. Training will be a lot faster and require less data [29]. In this study, we use two hidden layers due to their good ability to solve classification problems based on an ANN model [58].

In determining the number of input neurons, using too few neurons will result in underfitting, while using too many neurons will result in overfitting and taking longer to train the network [58]. As for the problem of determining the number of hidden layers, we can gradually increase the number of neurons until the network starts overfitting [29]. The rule-of-thumb approaches that we can practically apply for determining the correct number of neurons to use in the hidden layers [58], the number of hidden neurons should be: (a) between the size of the input layer and the size of the output layer, (b) 2/3 the size of the input layer plus the size of the output layer, and (c) less than twice the size of the input layer. Thus, in this study, we determined the number of neurons in hidden layers based on the number of nodes in input and output layers, as shown in Table 5.

The activation function is used to compute the weighted sum of input (*z*) and biases and to decide whether a neuron can be activated. The activation function takes various forms, i.e., linear or non-linear. Non-linear activation functions are mainly divided based on their range or curves. This study proposed two non-linear activation functions: ReLU (Rectified Linear Unit) and Softmax. The ReLU was used for two hidden layers, while the softmax was for the output layer. ReLU maximizes the negative value z to become 0 and allows the positive value z as the given value [40]. Softmax in the output layer is chosen because it can predict a multinomial probability distribution. Hence, this activation function is appropriate for overcoming multiclass classification problems. Class membership requires more than two class labels. Therefore, the target variable comprises the class label encoded using the one-hot encoding technique.

The nodes in the MLP model are composed of inputs and weights used to calculate a weighted sum of the inputs. The kernel initializer, also known as weight initializer, has the main task of initializing the weights of a neural network. To function properly, the variance of the outputs of each layer ought to be equal to the variance of its inputs, and the gradients must have equal variance before and after flowing through a layer in the reverse direction. It is actually impossible to guarantee both unless the layer has an equal number of inputs and neurons [29]. Several papers [53,54,59,60] provided methods for initializing weights for different activation functions with mathematical details. According to Geron in his book [29], initialization parameters based on types of activation functions for ReLU and softmax can be Glorot initialization and He initialization, respectively, with a uniform distribution.

Feedforward neural networks are ANNs that connect inputs with outputs, see Figure 1. This one-direction calculation generates the predicted value (*ŷ*) in the output layer. The loss function is used to find errors or deviations in the learning process by comparing and measuring the disagreement between the actual (*y*) and predicted (*ŷ*) output values. Since we used one-hot encoding in the output class and transformed the class to categorical data, we decided to use the ‘*categorical_crossentroy’* loss function. The backpropagation algorithm allows the computation of loss gradients with respect to updating the weights and bias using the chain rule [61]. If the loss is still high, the feedforward and backpropagation processes will continue until it generates a small value indicating that the predicted values are very close to the actual values. The calculation only occurred once, passed forwardly and backwardly through the ANNs, called one epoch [40]. The process of reducing the loss function to a minimum is called optimization. The network will learn (iterative and incremental updates on weights) patterns that can correctly predict a given input sample to the correct output [61]. The optimizer takes action to update the weights by optimizing the learning process of ANNs. The optimizer will stop when it achieves optimal results in learning [40]. Adam (adaptive moment estimation), as we used in this study, is the most popular choice recently for optimization in deep learning with excellent and rapid results [40,61]. This approach combines the best ideas of stochastic gradient descent, specifically AdaGrad and RMSProp. A paper by Kingma and Ba [62] provides explanations with mathematical procedures and proves that Adam has shown good performance in optimizing multilayer neural networks. The metric of ‘accuracy’ was applied in the compilation step to evaluate the performance of the MLP model as we develop this model to solve the classification problem.

Lastly, we must define the number of epochs and the batch size to train our MLP model. One epoch means that the overall dataset is passed forward and backward through the network only once. Because this process affects updating weights, using one epoch or single pass is insufficient. One epoch leads to underfitting. As the number of epochs increases, this will lead from underfitting to optimal to overfitting. It is required to control the number of epochs by using the ‘EarlyStopping’ callback function. The training will stop when a monitored metric has stopped improving. The metric value of ‘validation loss’ was used in this study as a quantity to be monitored. Training tends to stop when there is an increment in loss values. A total of 100 epochs were used in the algorithm as a maximum control point. The number of epochs with no improvement after the training stops, or called patience, was determined to be 5. Batch size is the number of sample data points in a single batch. The number of iterations equals the number of single batches required to complete one epoch. As we had 2400 data points and the batch size was determined to be 100, it took 24 iterations to complete one epoch.

**Figure 7:**
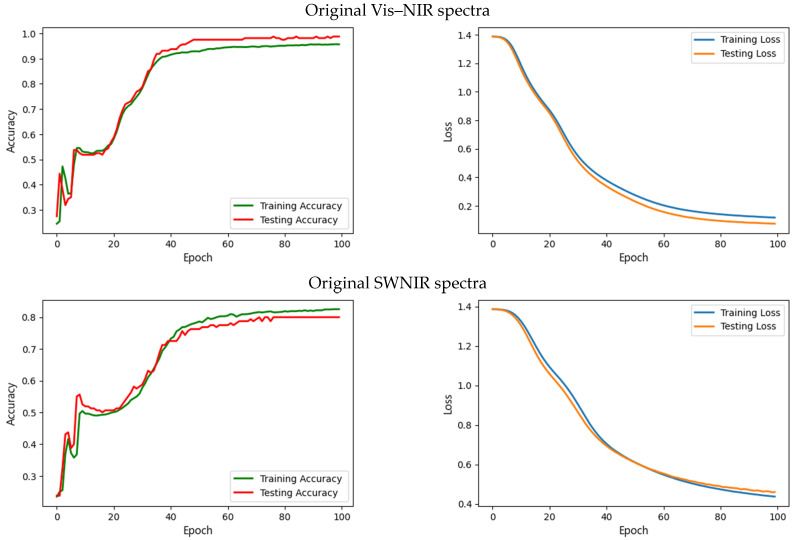
Model accuracy and loss curves.

Figure 7 shows the accuracy and loss during the learning process over the number of epochs. The accuracy curve figures out how well the model classifies the samples by comparing the predicted and actual classes. The loss curve depicts the error in classifying the samples. These two curves also diagnose issues during the learning process that can lead to underfitting and overfitting. Overfitting indicates that the model performed well in training data but poorly in testing new data. Underfitting means that the model can neither train data nor test new data. To overcome these issues, since we control validation errors during the learning, ‘EarlyStopping’ tracks model parameters/weights and then halts the learning after the best performance so far over the validation set does not improve over increasing epochs [61]. Thus, all curves represent the number of epochs (*x*-axis) varied. However, it is good to plot loss and accuracy across epochs instead of iteration because it will be calculated across every data item (and give the quantitative loss/accuracy at the given epoch) rather than over the entire dataset.

Figure 7 signifies the loss and accuracy curves of training and testing, providing information on changes in the performance of the classifier learning process over the number of epochs. The loss curve represents the summation of errors in our model’s learning. If the error is high, the loss will be high, too; the learning process shows inadequate performance. The accuracy curve examines how well the model classifies the samples by comparing the model’s predicted results with the actual class. High accuracy indicates that the model produces minor errors in the learning process. These two curves can also diagnose any issues with the learning process that could be causing underfit or overfit models. Following Figure 7, the MLP model was performed with high accuracy and low loss. High accuracy means the curve tends to closely reach 1 (or 100%), while at a low loss, the curve decreases significantly and tends to flatten as it approaches 0. The loss and accuracy of the Vis–NIR spectra produce more significance in training the data than the SWNIR spectra. It was demonstrated from the loss curve that samples from Vis–NIR spectra tend to flatten when they reach epoch 80. It can be implied that the MLP works better and produces superior performance in training samples acquired with Vis–NIR spectra.

### 3.4. Model Performance

The MLP model’s performance was evaluated based on performance metrics and ROC-AUC. The results of the matrices in Table 6 and Table 7 were calculated using the confusion matrix for the given dataset—training and testing sets. The confusion matrix was calculated from two sets of spectral data—VisNIR and SWNIR—and the spectral preprocessing algorithm applied. Because we used the *k*-fold cross-validation method to examine the classification success of the model objectively, the training set was divided into training and validation folds. As we determined the *k* value as 10, the *k* − 1 folds were reserved for training, the one fold for validation, and the *k* number of iterations. This validation process proceeded until each part of the data was used and the process was repeated *k* times (10 iterations) [41]. The model validation process produced accuracies of more than 80%. 

The confusion matrix values (*TP*, *TN*, *FP*, and *FN*) will be examined to obtain performance evaluation parameters, such as accuracy, specificity, precision, recall, F-score, and misclassification error. These parameters will enable us to comprehend how well the model determines the classification process with the given data. The statistical results were performed in average values as listed in Table 6 and Table 7. The results clearly showed that the MLP model achieved the best performance in the classification process of coffee origins, where the average values of accuracy for both spectrometers and spectral processing algorithms applied reached >90%, the error generated was lower than 10% and had values close to 1.00 for specificity, precision, recall, and F-score.

**Figure 8:**
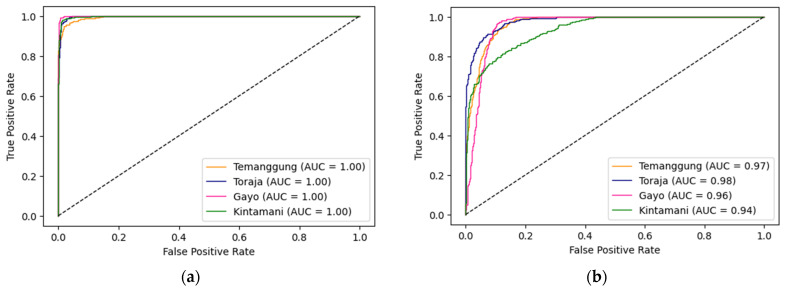
ROC-AUC from original spectra (**a**) Vis–NIR and (**b**) SWNIR.

The ROC (Figure 8) is a graphic plot that visualizes a classifier’s performance and tells us how the model can distinguish between classes. The curve plots the true positive rate (TPR, another name for recall) against the false positive rate (FPR). The FPR is the ratio of negative samples correctly classified as positive, or equal to 1–TNR (true negative rate). The TNR, also called specificity, is the ratio of negative samples that are correctly classified as negative. Hence, the ROC curve plots sensitivity (or recall) versus 1—specificity [29]. The model determines “bad” if the curve is close to the baseline that crosses from point (0,0) and determines “good” if the curve is close to point (0,1). As we graph the dotted line (the ascending diagonal of an ROC graph), a good classifier stays as far away from above that line as possible (toward the top-left corner) [29]. According to Figure 8, spectroscopic data from the Vis–NIR spectrometer were suitable as input data in the MLP model due to its ability to generate AUC equals to 1. 

AUC is a single number or percentage of area under the ROC curve, ranging between 0 and 1, and evaluates the ranking regarding the separation of the multiple classes. The higher the AUC value, the better the classifier’s performance. A perfect classifier will have a ROC-AUC equal to 1 [29]. Figure 6 demonstrates the higher AUC value representing the better performance of the classifier for four Arabica coffees. The AUC values were 1.00 for all samples at Vis–NIR spectra (Figure 8a), showing that the model could correctly distinguish between all positive and negative classes. A significant change in Figure 8b from SWNIR spectra implied that the model could distinguish positive class values from negative ones. Nevertheless, the model could still predict more true positives and true negatives rather than false negatives and false positives.

## 4. Discussion

Spectroscopic data contain multiple variables that may have large amounts of information and multicollinearity [63]. Reduction of dimensionality is required to map those variables which are a high dimensionality to a lesser dimensionality [64]. In many spectroscopic studies, an unsupervised learning algorithm which is also one of the prominent dimensionality reduction techniques in chemometrics analysis, is principal component analysis (PCA). PCA converts and summarizes spectroscopic data (a group of correlated variables) by forming new (uncorrelated) variables called principles components (PCs), which are linear combinations of the original variables [52,64]. 

Chemometric methods became routinely applied tools to handle problems related to spectroscopic data, including (a) determination of the concentration of a compound in a sample, (b) classification of the origins of samples, and (c) recognition of the presence/absence of substructures in the chemical structure of an unknown organic compound in samples. Currently, the chemometric approach is not only based on methods to solve chemical problems but also is data-driven, which can be applied to solve problems for other disciplines such as econometrics, sociology, psychometrics, medicine, biology, image analysis, and pattern recognition. This method uses multivariate statistical data analysis to analyze and restructure datasets, as well as to make empirical mathematical models that can predict the values of important properties that are not directly measurable [65]. Principal component regression (PCR) and partial least-square regression (PLSR) are methods to deal with calibration problems, while the classifications are discriminant analysis (DA), SIMCA, classification tree (CT), support vector machine (SVM), and machine learning algorithm including artificial neural networks (ANN).

The use of an ANN model in spectroscopic research has been found, including in identifying functional groups and qualitative analysis [66]. The backpropagation (BP) algorithm is commonly used to train feedforward neural networks that have only inter-layer connections and are fully connected from the input layer to the hidden layers and to the output layers. Here, the spectral information is used as input variables, and the analyte concentration, physical-chemical characteristic, or desired group of samples is used as output. However, the disadvantage of the ANN model is related to the complex infrastructure. To get robust learning results, it must use a large number of training samples. The input variables must also be higher than the number of outputs estimated. In spectroscopic data, the large number of spectral variables often renders the predicted outputs, but methods of reducing the variable dimensionality are often required so the model can work easier and take a few times to train the model [67]. 

The combination of PCA and ANN in the classification problems was demonstrated by He et al. [52] to discriminate the five typical varieties of yoghurt by Visible/NIR-spectroscopy (325 to 1075 nm). The first seven principal components (PC1 to PC7) from original spectra gained 99.97% of explained variance and are applied as input variables for BP-ANN. The distinguishment of five yoghurt types was performed satisfactorily. Briandet et al. [68] used ANN to detect adulteration in instant coffee using infrared spectroscopy. Five types of samples were determined, including pure coffee, coffee + glucose, coffee + starch, and coffee + chicory. The ANN model output was an improvement over the classification results obtained by LDA.

A spectrum with a large number of variables is not recommended to be used directly as an input variable for an ANN and should be compressed first [66]. PCA can be applied to compress a large number of spectral data into a small number of variables defined as PCs. These small variables represent the most common data variations that can be attributed to the first, one, two, or three components and soon. These components can replace the original spectroscopic variables without much loss of information [69]. To obtain new data, generally, many scientists determine the number of PCs that explain more than 85% of the cumulative variance ratio [21,52,65]. Practically, a value greater than 85% is not always necessary to be achieved. The number of PCs obtained can also be changed to any extent according to the circumstance [52]. There is no definitive answer to the question of how many the number of components to retain. It depends on the amount of total cumulative variance explained, the relative size of the eigenvalues (the variances of the sample components), and the subject-matter interpretation of the components [69]. Referring to [68], we can consider the number of components by excluding eigenvalues near zero because they are deemed unimportant and may indicate an unsuspected linear dependency in the data. Therefore, in this study, we use PCs with an explained variance ratio greater than 0.5% to overcome this issue. According to Table 4, the number of components to be included in the model varies.

In special cases for calibration problems, applying PCA in data compression may run the risk of ignoring some useful information correlated to the analytes, a relatively large number of PCs should be used. Approaches for future studies in the classification problems can be carried out by employing ANN not only combined with PCA but also with LDA and PLS regression. LDA compresses spectroscopic data and produces less-dimensional variables called linear discriminants (LDs). Spectral information also can be compressed through a PLS regression, and the PLS factors were used as input for the ANN model [65,70]. 

## 5. Conclusions

To ensure food safety and satisfaction, spectroscopic methods have emerged and become a powerful technique in examining the chemical composition, quality, and authenticity of food and agricultural products, including in the coffee industry. Efficient chemometric analysis and machine learning models are required to obtain the best results. This work showed an accurate and non-destructive approach for authenticating the origins of agro-products. The combination of PCA and MLP was established and a superior classification process was conducted for Arabica coffee beans. PCA extracted the important information from spectroscopic data and visualized the information in a low-dimensional space, called PCscore. The MLP used the positions of samples in this space as input variables. The performance results confirmed that the MLP model integrated with PCA has proven to be superior, suitable, and successful for verifying the origin of Arabica coffee.

## Figures and Tables

**Table 1:** Four-classes confusion matrix.

		Predicted Classes			
		*C*_1_	*C*_2_	*C*_3_	*C*_4_
Actual classes	*C*_1_	*T*_1_	*F*_12_	*F*_13_	*F*_14_
	*C*_2_	*F*_21_	*T*_2_	*F*_23_	*F*_24_
	*C*_3_	*F*_31_	*F*_32_	*T*_3_	*F*_34_
	*C*_4_	*F*_41_	*F*_42_	*F*_43_	*T*_4_

**Table 2:** Determination of TP, TN, FP, and FN.

Class	*TP*	*TN*	*FP*	*FN*
*C*_1_	*T*_1_	*T*_2_ + *T*_3_ + *T*_4_ + *F*_23_ + *F*_24_ + *F*_32_ + *F*_34_ + *F*_42_ + *F*_43_	*F*_21_ + *F*_31_ + *F*_41_	*F*_12_ + *F*_13_ + *F*_14_
*C*_2_	*T*_2_	*T*_1_ + *T*_3_ + *T*_4_ + *F*_13_ + *F*_14_ + *F*_31_ + *F*_41_ + *F*_34_ + *F*_43_	*F*_12_ + *F*_32_ + *F*_42_	*F*_21_ + *F*_23_ + *F*_24_
*C*_3_	*T*_3_	*T*_1_ + *T*_2_ + *T*_4_ + *F*_12_ + *F*_14_ + *F*_21_ + *F*_24_ + *F*_41_ + *F*_42_	*F*_13_ + *F*_23_ + *F*_43_	*F*_31_ + *F*_32_ + *F*_34_
*C*_4_	*T*_4_	*T*_1_ + *T*_2_ + *T*_3_ + *F*_12_ + *F*_13_ + *F*_21_ + *F*_23_ + *F*_31_ + *F*_32_	*F*_14_ + *F*_24_ + *F*_34_	*F*_41_ + *F*_42_ + *F*_43_

**Table 3:** Performance metrics.

Metrics	Formula		Metrics		Formula
Accuracy	*AC*	$=\frac{TP+TN}{TP+FP+TN+FN}$	Precision	*P*	$=\frac{TP}{TP+FP}$
Misclassification error	*E*	$=\frac{FP+FN}{TP+FP+TN+FN}$	Recall or sensitivity	*R*	$=\frac{TP}{TP+FN}$
Specificity	*SP*	$=\frac{TN}{TN+FP}$	F-Score	*FS*	$=\frac{2\cdot R\cdot P}{\left(R+P\right)}$

**Table 4:** Explained variance ratio of PCA.

		Explained Variance Ratio (%)							Number of PC
		1	2	3	4	5	6	7	
Vis-NIR									
	Original	91.73	3.38	1.35	0.53				4
	SMA	97.16	1.84	0.54					3
	SGS	94.26	3.38	1.29					3
	SNV	37.18	17.57	5.46	4.83	2.74	2.24	1.66	7
	MSC	37.46	17.58	5.39	4.81	2.71	2.23	1.65	7
	SG-1D	59.50	15.02	4.74	3.76	2.44	2.27	1.47	7
SWNIR									
	Original	99.05	0.54						2
	SMA	99.26	0.41						2
	SGS	99.06	0.54						2
	SNV	58.67	28.23	5.65	3.06	1.81	0.83		6
	MSC	58.74	28.22	5.64	3.06	1.81	0.83		6
	SG-1D	86.74	9.56	1.47	0.53				4

**Table 5:** Structure of the MLP model.

Parameters	Input Layer				Hidden Layer 1	Hidden Layer 2		Output Layer/Label	
			Vis-NIR	SWNIR					
Number of neurons	Ori		4	2	6	6		4	class 0: temanggung
	SMA		3	2					class 1: toraja
	SGS		3	2					class 2: gayo
	SNV		7	6					class 3: kintamani
	MSC		7	6					
	SG-1D		7	4					
Activation Function					ReLU	ReLU		Softmax	
Weight initialization					glorot_uniform	glorot_uniform		he_uniform	
Loss function	:	Categorical cross-entropy			Max. epochs to train		:	100	
Optimizer	:	Adam			Batch size		:	100	
Validation control	:	Metric ‘Accuracy’			Callback Function		:	EarlyStopping	

**Table 6:** Averaged values of performance matrices—Vis–NIR spectra.

Spectra	Metrics						Cross-Validation
	AC *	SP	P	R	FS	E *	Mean ± SD Accuracy *
Training							
Original	97.89	0.99	0.96	0.96	0.96	2.11	95.15 ± 2.31
SMA	98.57	0.99	0.97	0.97	0.97	1.43	95.46 ± 6.82
SGS	96.51	0.98	0.93	0.93	0.93	3.49	92.04 ± 2.39
SNV	99.72	1.00	0.99	0.99	0.99	0.28	99.25 ± 0.54
MSC	99.69	1.00	0.99	0.99	0.99	0.31	98.75 ± 4.87
SG-1D	98.88	0.99	00.98	0.98	0.98	1.12	95.90 ± 3.80
Testing							
Original	98.30	0.99	0.97	0.96	0.97	1.70	
SMA	98.67	0.99	0.97	0.97	0.97	1.33	
SGS	96.46	0.98	0.93	0.93	0.93	3.54	
SNV	99.62	1.00	0.99	0.99	0.99	0.38	
MSC	99.81	1.00	1.00	1.00	1.00	0.19	
SG-1D	98.93	0.99	0.98	0.98	0.98	1.07	

* units in percentage (%).

**Table 7:** Averaged values of performance matrices—SWNIR spectra.

Spectra	Metrics						Cross-Validation
	AC *	SP	P	R	FS	E *	Mean ± SD Accuracy *
Training							
Original	91.11	0.94	0.82	0.83	0.82	8.89	83.33 ± 3.41
SMA	96.70	0.98	0.93	0.93	0.93	3.30	92.04 ± 1.92
SGS	91.95	0.95	0.84	0.84	0.84	8.05	84.45 ± 2.00
SNV	97.54	0.98	0.95	0.95	0.95	2.46	94.34 ± 1.45
MSC	97.85	0.99	0.96	0.96	0.96	2.15	92.54 ± 6.57
SG-1D	98.54	0.99	0.97	0.97	0.97	1.46	96.27 ± 1.63
Testing							
Original	90.59	0.94	0.81	0.82	0.81	9.41	
SMA	95.83	0.97	0.92	0.92	0.92	4.17	
SGS	90.72	0.94	0.81	0.82	0.81	9.28	
SNV	97.47	0.98	0.95	0.95	0.95	2.53	
MSC	97.22	0.98	0.94	0.94	0.94	2.78	
SG-1D	98.36	0.99	0.97	0.97	0.97	1.64	

* units in percentage (%).

## Data Availability

The data presented in this study are available on request from the corresponding author.
